# Molecular Modeling of Antimalarial Agents by 3D-QSAR Study and Molecular Docking of Two Hybrids 4-Aminoquinoline-1,3,5-triazine and 4-Aminoquinoline-oxalamide Derivatives with the Receptor Protein in Its Both Wild and Mutant Types

**DOI:** 10.1155/2018/8639173

**Published:** 2018-06-24

**Authors:** Hanine Hadni, Mohamed Mazigh, El'mbarki Charif, Asmae Bouayad, Menana Elhallaoui

**Affiliations:** Engineering Materials, Modeling and Environmental Laboratory, Faculty of Sciences of Dhar Elmehraz, Sidi Mohammed Ben Abdellah University, B.P. 1796, Atlas, Fes, Morocco

## Abstract

Modeling studies using 3D-QSAR and molecular docking methods were performed on a set of 34 hybrids of 4-aminoquinoline derivatives previously studied as effective antimalarial agents of wild type and quadruple mutant *Plasmodium falciparum* dihydrofolate reductase (DHFR). So, the famous mathematical method multiple linear regression (MLR) was explored to build the QSAR model. The DFT-B3LYP method with the basis set 6-31G was used to calculate the quantum chemical descriptors, chosen to represent the electronic descriptors of molecular structures. On the contrary, the MM2 method was used to calculate lipophilic, geometrical, physicochemical, and steric descriptors. The QSAR model tested with artificial neural network (ANN) method shows high performance towards its predictability. The predicted model was confirmed by three validation methods: leave-one-out (LOO) cross validation, Y-randomization, and validation external. The molecular docking study of three compounds **9**, **11**, and **26** on both wild and quadruple mutant types of pf-DHFR-TS as the protein target helps to understand more and then predict the binding modes with the binding sites.

## 1. Introduction

Malaria is one of the world's greatest global public health challenges. It is most prevalent in sub-African, Asian, and South American countries, and it mostly affects children under the age of five and pregnant women [1, 2]. According to a world health organization (WHO) report 2015, estimated 3.2 billion people were at risk of malaria, approximately 212 million cases of malaria worldwide, and 429.000 deaths occurred worldwide in 2015. Of these estimated deaths, 90% occurred in sub-Saharan Africa [1]. Malaria is an infectious and contagious disease caused by the protozoa of the genus *Plasmodium* [3]. There are five species that infect humans (*P. falciparum*, *P. vivax*, *P. malariae*, *P. ovale*, and *P. knowlesi*) [4]. However, amongst these five species, *Plasmodium falciparum* is the most severe and lethal species [5]. Many efforts are made in attempts to find out efficient inhibitors for this protein by testing several molecular structures. The quinoline moiety has attracted a great consideration of the medicinal chemists, as it is one of the crucial pharmacophores accountable for imparting antimalarial action [6, 7]. On the contrary, the 1,3,5-triazine derivatives cycloguanil, chlorcycloguanil, clociguanil, and WR99210 are already approved as effective dihydrofolate reductase (DHFR), specific inhibitor of *P. falciparum* domain, and they selectively inhibit biochemical processes that are vital for parasite growth [8]. Nowadays, to overcome drug resistance problems the concept of hybrid molecules has been introduced, in which two or more pharmacophores are linked together (as quinoline-triazine and qunoline-oxalamide), and it is believed that these compounds act by inhibiting simultaneously two conventional targets [9]. In this study, we worked on these two pharmacophores, as two types of hybrids: 4-aminoquinoline-triazine and 4-aminoquinoline-oxalamide [10].

The discovery of new antimalarial drugs is very challenging; the aim of developing a QSAR model is to construct a relationship (using statistical methods) between structural properties and activities using a training set which is capable of predicting the activity of compounds which are not used to build the model by multiple linear regression (MLR) and artificial neural network (ANN) calculations. The QSAR model has been validated by using an internal and external validation as well as Y-randomization. To develop the binding modes of this set of hybrids in the active sites, we have to perform the docking of three compounds: on the one hand, the highest active compound **26** belonging to the triazine series and the highest active compound **9** from the oxalamide series and on the other hand, the lowest active of the entire series compound **11**, with *Plasmodium falciparum* dihydrofolate reductase–thymidylate synthase (pf-DHFR-TS) in its two forms: the wild type and the quadruple mutant [11]. This study allows the developing of models that not only provide details of the binding modes and key molecular interactions but also allow the prediction of relative inhibition and binding affinities that could be reproduced in silico.

## 2. Materials and Methods

### 2.1. Experimental Data

**Figure 1:**
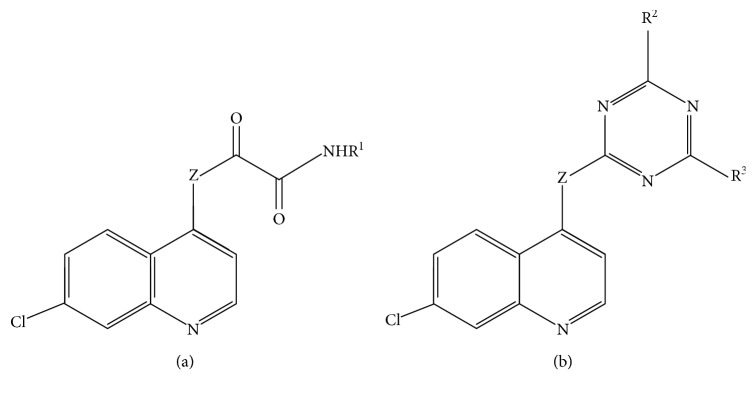
The general structure of 4-aminoquinoline-oxalamides (a) and 4-aminoquinoline-triazines (b).

In this work, a data set of 34 hybrids of 4-aminoquinoline [10] constituting two groups is explored. The first group (4-aminoquinoline-oxalamides) accounts for 16 molecules numerated from 1 to 16, and the second group (4-aminoquinoline-triazines) contains **18** compounds numerated from **17** to **34** (Figure 1). The chemical structures of these hybrid derivatives with their antimalarial activities (IC_50_) are presented in Tables 1 and 2. The observations are converted into logarithm scale log IC_50_.

### 2.2. Molecular Descriptors Calculation

In order to accurately model and predict inhibitors activities, 16 descriptors listed in Table 3 were introduced. Eleven descriptors which are lipophilic, geometrical, physicochemical, and steric descriptors were calculated with the MM2 method with the aid of the ACD/ChemSketch program [12] and the ChemBioOffice software [13]. On the contrary, 5 electronic descriptors were calculated with the DFT method [14], using the Gaussian03 quantum chemistry package [15]. The optimization of compounds was performed with the DFT method using Becke's three-parameter hybrid function (B3LYP) [16], with a 6-31G basis set in the case of electronic descriptors calculation and with the MM2 method for the remaining descriptors. The totality of descriptors used in this work is represented in Table 3.

### 2.3. Analysis Methods

Multiple linear regression (MLR) [17] analysis with the descendent selection method was used to select the most appropriate descriptors. It is a mathematical technique to study the relation between one dependent variable and several independent variables. The regression method is based on three criteria: correlation of determination (*R*^2^), the Fisher ratio value (*F*), and the root mean square error (RMSE). The MLR model was generated using the software XLSTAT version 2013 [18]. Note that the MRL has been served to select the used descriptors as the input parameters in the artificial neural network (ANN).

The ANN analysis is performed using the SAS JMP package (v8.0, SAS Institute Inc., Cary, NC, USA). The neurons networks are arranged in three layers: The input layer contains six neurons representing the relevant descriptors obtained with the MLR technique, the output layer contains one neuron representing the calculated activities values log IC_50_, and the hidden layer is composed of 3 neurons determined by *ρ* = (number of weight)/(number of connection). In this work, we used the *ρ* value interval 1 < *ρ* < 3 [19, 20].

The high correlation coefficient indicates the quality of the equation that fit the data, in order to explore the stability of this equation; the cross-validation method with “leave-one-out” was carried out using the ANN method. Based on this technique, a number of modified data sets are created by deleting in each case one individual [21], and thereafter, the corresponding models serve to predict the activity of the removed compound.

The LOO cross-validation coefficient *R*^2^ was calculated as follows [22]:

(1) $$\begin{array}{c} {\text{Rcv}}^{2}=1-\frac{\sum_{i=1}^{n}{\left(Y_{\exp}-Y_{\text{pred}}\right)}^{2}}{\sum_{i=1}^{n}{\left(Y_{\exp}-Y\right)}^{2}}, \end{array}$$

where *Y*_exp_ and *Y*_pred_ are the observed and predicted values for the dependent variables, respectively, and *Y* is the average observed value.

In order to ensure the reliability of the QSAR model, the Y-randomization test has been used. This approach consists to randomly mix many properties/experimental activities for the learning series using the same descriptors; the new QSAR model is constructed to exclude the possibility of random correlation in the obtained model [23].

Furthermore, external validation is necessary as the validation method is used to ensure the ability of the QSAR model. However, the data set in this work has been randomly divided into a training set with **28** compounds for the model developed through MLR, and a predicted set with **6** compounds has been reserved to external validation.

The ability of the built model based on the external prediction set was evaluated by *R*_ext_^2^, which could be calculated as follows [22]:

(2) $$\begin{array}{c} R_{\text{ext}}^{2}=1-\frac{\sum {\left(Y_{\text{pred}\left(\text{test}\right)}-Y_{\left(\text{test}\right)}\right)}^{2}}{\sum {\left(Y_{\text{test}}-\bar{Y_{\text{tr}}}\right)}^{2}}, \end{array}$$

where *Y*_pred(test)_ and *Y*_(test)_ are the predicted and experimental values of the samples for the prediction set, respectively. $\bar{Y_{\text{tr}}}$ is the average value for the dependent variable for the training set.

The value of *R*_ext_^2^ ≥ 0.5 is considered as an indicator of the reliability of the model. However, Golbraikh and Tropsha showed that *R*_ext_^2^ is not a good parameter to estimate the reliability of the QSAR model. Indeed, an external validation based on the Golbraikh and Tropsha criteria is necessary [22].

In order to gain insight into the key structural requirements of the antimalarial activity, molecular docking studies are carried out using the AutoDock4.2 program [24]. X-ray crystallography structures of *Plasmodium falciparum* of the wild type (coded as 1J3I.pdb) and quadruple mutant (coded as 1J3K.pdb) pf-DHFR-TS were obtained from the Protein Data Bank [25]. The minimized protein structures were defined as receptors, and the first step in the preparation of the receptor was the removal of the ligands and the water molecules. In order to simplify the docking analysis, in this docking, the 3D grid was created by the AUTOGRID algorithm [26] to evaluate the interacting energy between protein ligands. The grid maps were constructed using 60, 60, and 60, pointing in *x*, *y*, and *z* directions, with grid point spacing of 0.375 Å. The center grid box is of 29.39 Å, 5.56 Å, and 52.49 Å, by the ligand location in the complex. Discovery Studio software was used for the 2D and 3D visualizations of the established interactions [27].

## 3. Results and Discussion

In this study, we used two random distributions of compounds into the training and test sets. The first training set included **28** compounds, and the corresponding test set included **6** compounds. The selected descriptors values, and predicted activities values using the training set obtained by MLR, ANN, and CV methods, are summarized in Table 4.

### 3.1. Multiple Linear Regression

**Figure 2:**
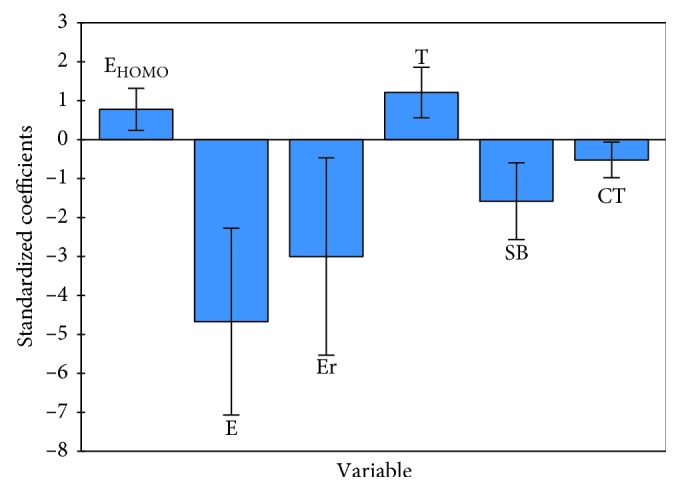
Modeling characterization by the normalized coefficients.

**Figure 3:**
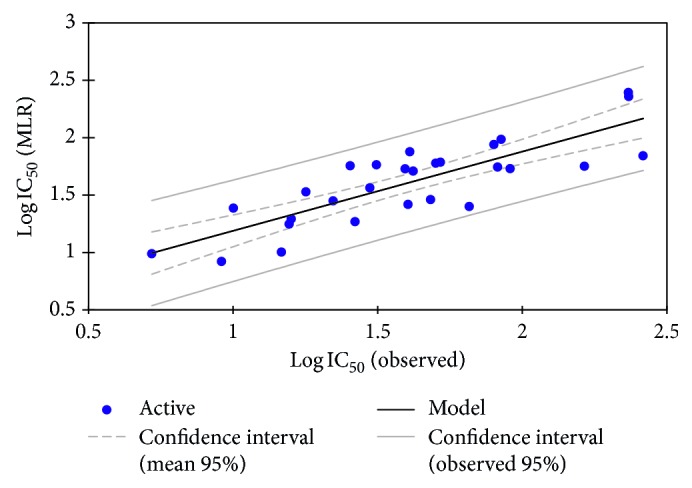
Correlation of observed and predicted activities calculated using the MLR method.

The QSAR model of the training set built using the MLR method is represented by the following equation:

(3) $$\begin{array}{c} \log\;\;{\text{IC}}_{50}=-17.79+2.54\;*\;E_{{\text{HOMO}}^{-}},\;\;3E^{-4}\;*\;E-0.8E^{-4}\;*\text{ Er}+0.146\;*\;T-3.47\;*\text{ SB}-6.34E^{-3}\;*\text{ CT,}\\ N=28,\;\;R=0.83,\;\;R^{2}=0.69,\;\;F=57.86,\;\;\mathrm{RMSE}=0.27, \end{array}$$

where *N* is the number of compounds, *R* is the correlation coefficient, *R*^2^ is the determination coefficient, RMSE is the root mean square error, and *F* is the Fisher test. The relevant descriptors involved in the MLR model are HOMO energy, total energy, repulsion energy, torsion, critical temperature, and stretch-bend. The corresponding normalized coefficients are presented in Figure 2, and the correlation of the observed activities with the MLR calculated ones is illustrated in Figure 3.

As indicated by the statistical coefficient values of the correlation between the observed and calculated activities based on this model using the training set are quite significant, and the low RMSE indicates that the model is reliable to a better prediction precision.

### 3.2. Artificial Neural Networks

**Figure 4:**
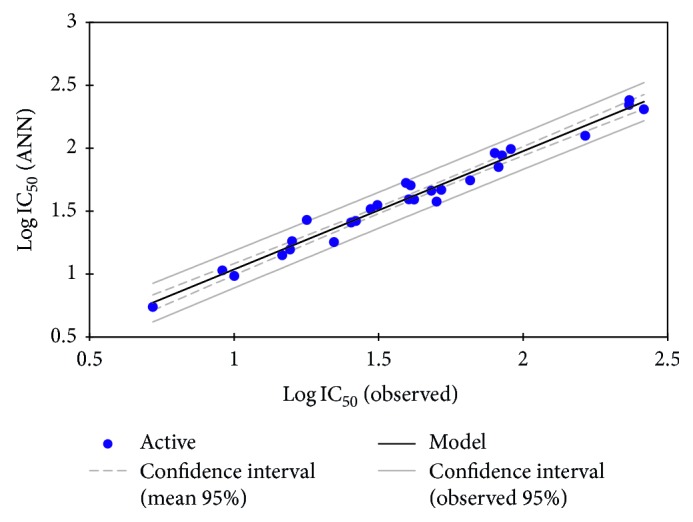
Correlation of observed and predicted activities calculated using ANN.

In order to increase the probability of good characterization of studied compounds, artificial neural networks (ANN) are used as the nonlinear method to generate predictive nonlinear model between observed antimalarial activities values and the set of molecular descriptors obtained by MLR with that of the architecture network (6-3-1). The correlation of the observed activities with the ANN predicted ones is illustrated graphically in Figure 4.

As it is shown in Figure 4, a good correlation between observed antimalarial activities values and predicted activities by ANN is obtained, in fact the correlation coefficient *R* = 0.98, the determination coefficient *R*^2^ = 0.97, and the standard error of estimate RMSE = 0.09. Such results show that the selected descriptors by MLR are pertinent, the ANN model possesses a significantly statistical quality, and the model proposed to predict antimalarial activity is relevant.

### 3.3. Cross Validation

**Figure 5:**
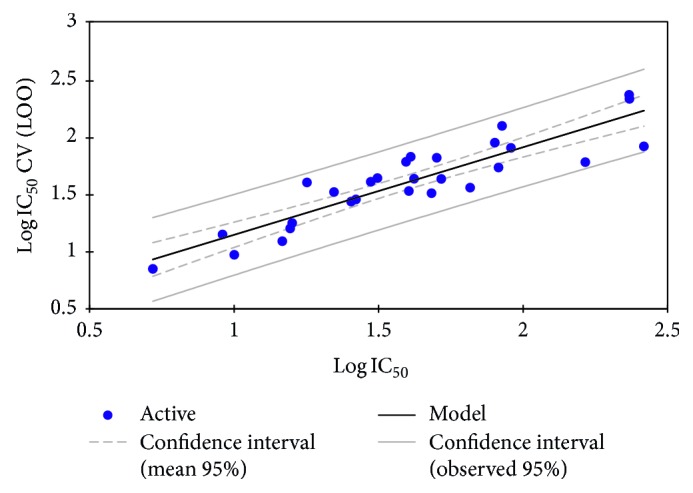
Correlation of observed and predicted activities calculated using CV (LOO).

The QSAR model proposed to predict the activity of new compounds should be tested. To validate our results, we used the LOO procedure, which involves removing a single molecule from the set containing 28 molecules and making a prediction for antimalarial activity. This procedure is repeated 28 times in order to estimate the predictive ability of such models. The correlation of the observed activities with the calculated cross-validation ones is shown graphically in Figure 5.

The obtained correlation (*R* = 0.90, *R*^2^ = 0.81, and RMSE = 0.16) shows a high predictive power of the MLR model. This result shows that our QSAR model is not sensitive to this operation of putting a molecule aside and putting it back into the learning series. This is a first indication of the stability of the selected QSAR model.

### 3.4. Y-Randomization

**Figure 6:**
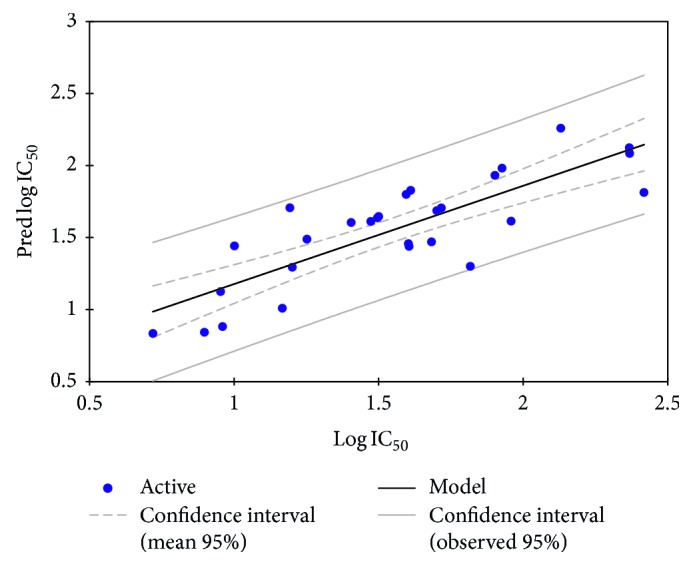
Correlation between observed/predicted by the Y-randomization test.

The Y-randomization test was performed to make sure that there is no random correlation. In this way, we could test the validity of the established QSAR model and check that the selected descriptors are not random, and consequently, the result model should have low statistical quality. The results of the Y-randomization method are given in Table 5 and Figure 6.

The new QSAR model built using the Y-randomization method is represented by the following equation:

(4) $$\begin{array}{c} \log\;\;{\text{IC}}_{50}=-13.6+2.32\;*\;E_{{\text{HOMO}}^{-}},\;\;4.5E^{-4}\;*\;E-0.9E^{-4}\;*\text{ Er}+0.141\;*\;T-2.43\;*\text{ SB}-9.33E^{-3}\;*\text{ CT,}\\ N=28,\;\;R=0.825,\;\;R^{2}=0.682,\;\;F=7.51,\;\;\mathrm{RMSE}=0.29. \end{array}$$

The correlation coefficient value of the mixture samples is close to that obtained by applying the model by the training set. This result provides the absence of dependence between descriptors included in the QSAR model.

### 3.5. External Validation

In a study on efficient methods of validation for QSAR models, Golbraikh and Tropsha showed that LOO methods are necessary but not sufficient, claiming that external validation is inevitable and proposed some criteria which would help to validate a QSAR model. This validation is done in two steps: validation of the model MLR have calculated new compounds which are not used in the model development of the training set (Table 6) and verification of the Tropsha criteria (Table 7).

The results show that Golbraikh and Tropsha criteria are successfully validated. All validations indicate that the built QSAR model is robust and satisfactory. The model established in this study meets all of the principles for QSAR validation and can be used to predict the antimalarial activity.

### 3.6. Docking Studies

**Figure 7:**
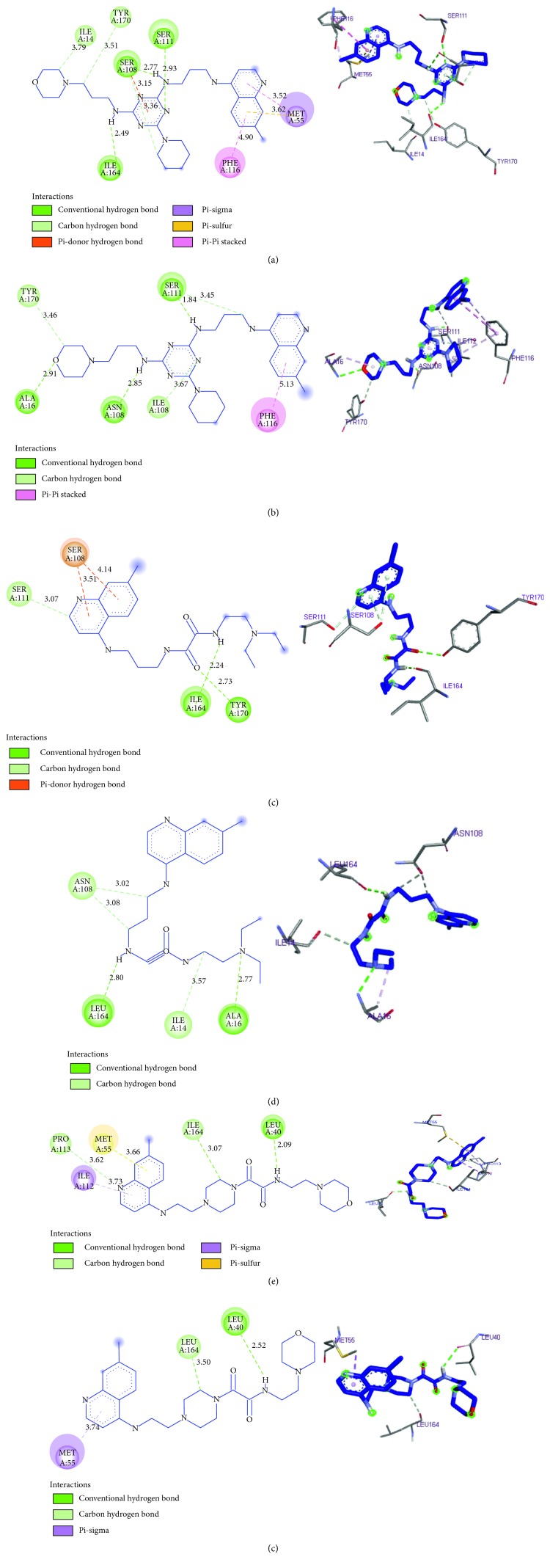
2D and 3D docking poses showing interactions of compounds **9**, **26,** and **11** in the binding sites of wild type and quadruple mutant of pf-DHFR-TS. (a) Compound **26**: wild type of pf-DHFR (binding energy −8.9 kcal/mol). (b) Compound **26**: quadruple mutant of pf- DHFR-TS (binding energy −8.3 kcal/mol). (c) Compound **9**: wild type of pf-DHFR (binding energy −7.42 kcal/mol). (d) Compound **9**: quadruple mutant of pf-DHFR-TS (binding energy −7.96 kcal/mol). (e) Compound **11**: wild type of pf-DHFR (binding energy −8.58 kcal/mol). (f) Compound **11**: quadruple mutant of pf-DHFR-TS (binding energy −9.2 kcal/mol)

In a pioneering study on the binding modes and the localization of the principal active sites in wild and mutant protein performed with a potent inhibitor 1,3,5-triazine derivative which is a preclinical molecule called WR99210, it is found that the important sites are located in Ile14, Ala16, Met55, Asp54, Ser108, Ile164, and Tyr170 in the case of the wild type and Ala16, Cys50, Asn51, Cys59, Asn108, Leu164, and Tyr170 in the case of mutant protein [28]. In a tentative to give insight into the interaction modes and to find out the interaction types established with this protein (pf-DHFR-TS) in its two forms, wild and mutant, the molecular docking study performed in this work is applied on three compounds **9** (IC_50_ = 15.58), **11** (IC_50_ = 261.84), and 26 (IC_50_ = 5.23) with the binding sites of both wild type and quadruple mutant. The docking results and docked conformations of ligands in the active sites are represented in Figure 7.

In the case of the wild type, compound **26** performs hydrogen bonding with the carboxylate oxygen atoms of ILE164, SER108, and SER111 by the involvement of the two NH groups bounded to the triazine group and one of the triazine nitrogen, with, respectively, the distances 2.49 Ǻ, 2.77 Ǻ, and 2.93 Ǻ, and a nonbonded p-sigma interaction between phenyl of quinoline with MET55 at a distance of 3.52 Ǻ. However, in the case of the quadruple mutant, three hydrogen bonds with ASN108, SER111, and ALA16 were observed by the involvement of two NH groups linked to 1,3,5-triazine and the oxygen of the morpholino group with a distance of 2.85 Ǻ, 1.84 Ǻ, and 2.91 Ǻ, respectively. For the compound **9**, two hydrogen bonds are formed between an oxygen and an azote of the oxalamide group with ILE164 and TYR170 with, respectively, the distances 2.24 Ǻ and 2.73 Ǻ in the case of the wild type. But in the case of the quadruple mutant, it forms two hydrogen bonds with ALA16 and LEU164 through the involvement of two azotes (the first is linked to the oxalamide group, and the second belongs to the diethylamine group), with distances of 2.77 Ǻ and 2.80 Ǻ, respectively. However, compound **11** showed only one hydrogen bonding interaction with LEU40 in both cases.

In the analysis of these results, we have at first observed that the residues with which the compounds **26** and **9** have formed their interactions are mentioned as the most important binding sites for antimalarial activity [28], which is not the case for compound **11**. Secondly, we observed that the number of hydrogen bonds differs from the most active compound which belongs to the triazine family, to the less active compound which belongs to the oxalamide family. So, this could explain the potent antimalarial activity for compound **26** and the importance of the triazine group to enhance the antimalarial activity compared to the oxalamide group.

## 4. Conclusion

The present study on a series of 4-aminoquinoline-triazines and 4-aminoquinoline-oxalamides was carried out using 3D-QSAR and docking techniques in the aim to predict the antimalarial activity. The group contribution method (for both training and test sets) was used to develop a reliable QSAR model for predicting antimalarial activity. The result of MLR and ANN methods using the training set clearly shows a strong relationship between the structural properties and the activity. Thus, the correlation coefficient for both methods shows good predictive ability of the model. The model is validated by internal and external validation methods including (leave-one-out) cross validation and Y-randomization. The obtained model shows good quality of the robustness to predicting the antimalarial activity. The observed activity was further corroborated via a molecular docking study which gave explanation to the differences observed among activities of compounds especially between the triazine family and oxalamide one. Results of these studies provided details of the predicted binding modes and the key molecular interactions. These will provide opportunities for medicinal chemists to develop new antimalarial drugs, by using new hybrid molecules.

## Figures and Tables

**Table 1:** Chemical structures and activities of 4-aminoquinoline oxalamide and derivatives.

Compounds	Structure of compound	Log IC_50_ (observed)
**1**	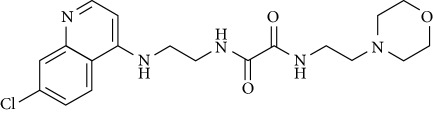	1.958

**2**	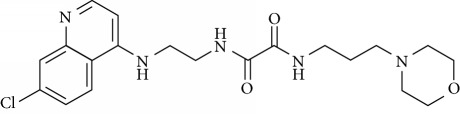	1.496

**3**	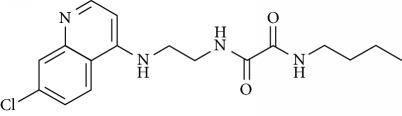	2.367

**4**	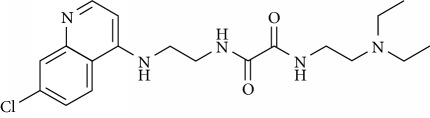	1.701

**5**	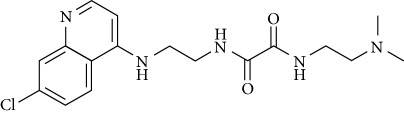	1.500

**6**	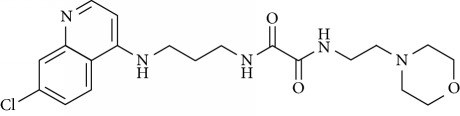	1.915

**7**	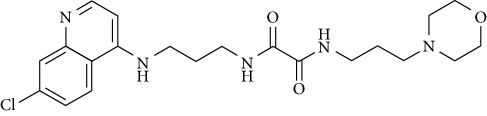	1.405

**8**	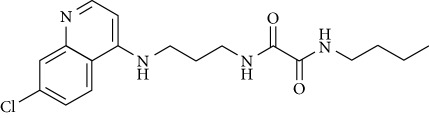	2.368

**9**	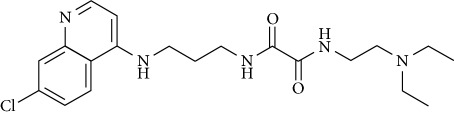	1.193

**10**	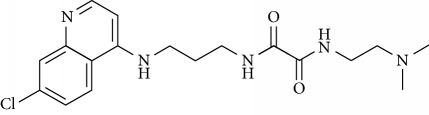	1.422
**11**	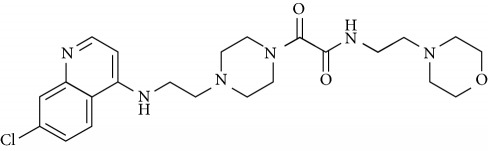	2.418

**12**	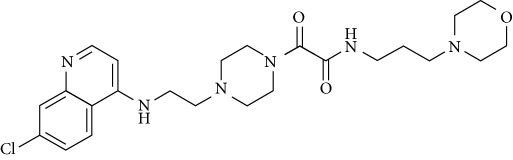	1.611

**13**	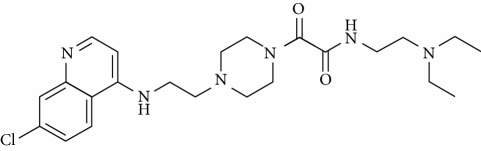	1.902

**14**	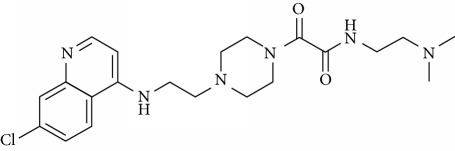	1.927

**15**	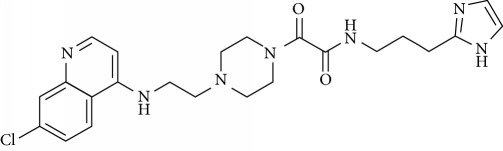	2.130

**16**	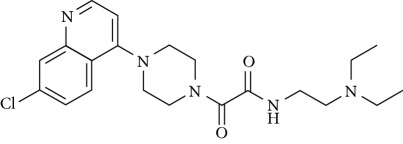	1.623

**Table 2:** Chemical structures and activities of the 4-aminoquinoline-triazine and derivatives.

Compounds	Structure of compound	Log IC_50_ (observed)
**17**	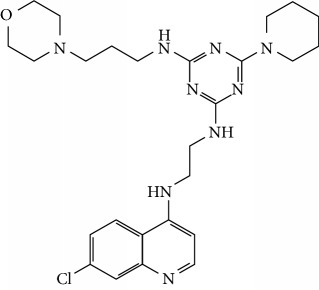	1.817

1**8**	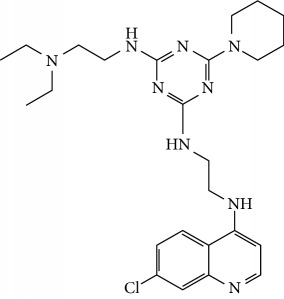	1.167

**19**	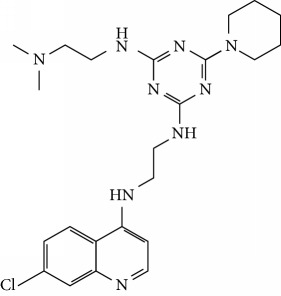	1.201

**20**	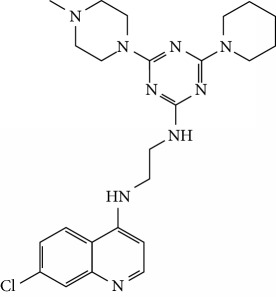	0.897
**21**	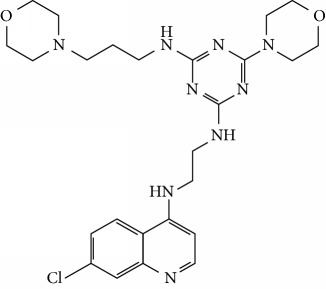	1.346

**22**	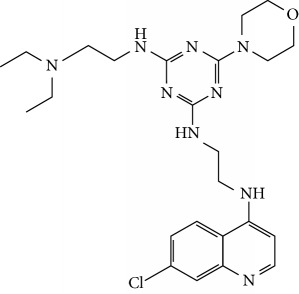	1.473

**23**	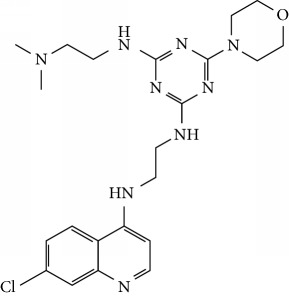	1.595

**24**	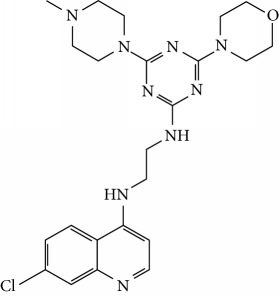	1.001
**25**	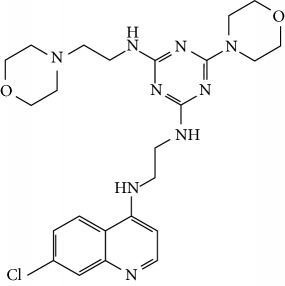	1.683

**26**	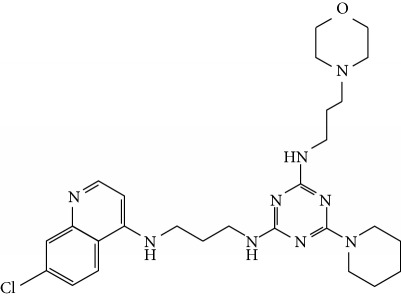	0.719

**27**	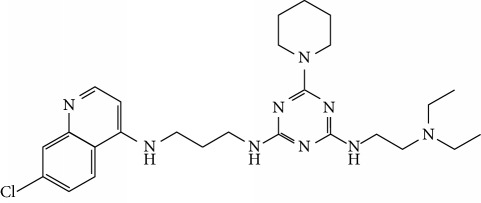	0.953

**28**	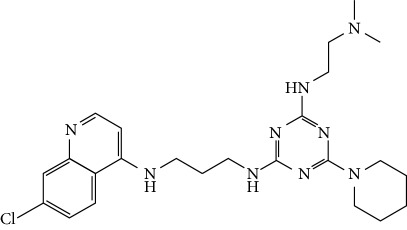	1.194

**29**	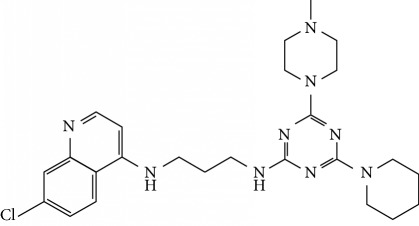	0.960
**30**	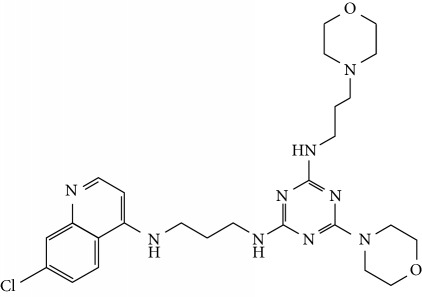	1.717

**31**	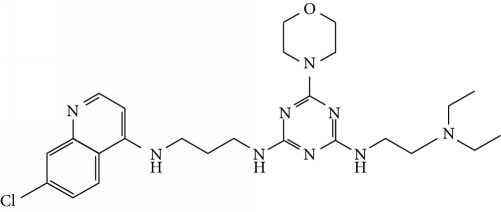	1.605

**32**	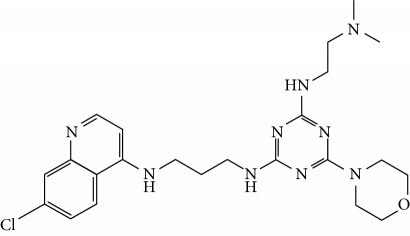	2.215

**33**	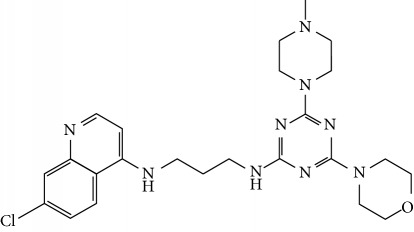	1.603

**34**	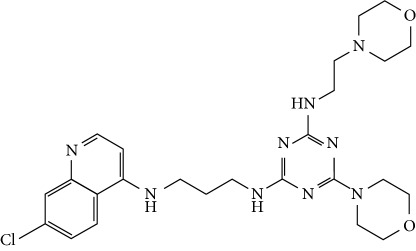	1.252

**Table 3:** List of descriptors constituting the database.

Category of descriptors	Electronic	Lipophilic	Geometrical	Physicochemical	Steric
Name of the descriptors	HOMO energy (E_HOMO_)	LogP (octanol-water partition coefficient)	Torsion (*T*)	Critical pressure (CP)	Density (*D*)
	LUMO energy (E_LUMO_)	VDW energy (E_VDW_)	Stretch-bend (SB)	Critical temperature (CT)	*D*=MW/MV
	Dipole moment (Dp)			Critical volume (CV)	Wiener index (*W*)
	Total energy (*E*)				Molar volume (MV)
	Repulsion energy (Er)				Number of H-bonding substituents (Hs)

**Table 4:** Values of the selected descriptors and the observed/predicted log IC_50_ values.

*N*	EHOMO	*E*	Er	*T*	SB	CT	Log IC_50_	MLR	ANN	CV (LOO)
1	5.496	−46195.5	66,323.4	−1.509	0.463	1170.7	1.958	1.730	1.993	1.901
2	5.447	−47264.9	68,848	−1.354	0.485	1179.5	1.496	1.763	1.547	1.638
3	5.848	−40538.9	52,297.9	−5.165	0.098	1107.9	2.367	2.393	2.343	2.363
4	5.531	−44182.8	63,712.2	−0.391	0.397	1134.3	1.701	1.777	1.575	1.815
6	5.459	−47265	69,290.9	−1.482	0.484	1179.5	1.915	1.744	1.849	1.730
7	5.41	−48334.4	71,918.9	−1.405	0.505	1189.3	1.405	1.754	1.408	1.434
8	5.764	−41608.5	55,210.6	−4.728	0.12	1115.3	2.368	2.358	2.381	2.328
10	5.709	−43113.3	58,873.4	−1.405	0.505	1189.3	1.422	1.268	1.421	1.452
11	5.385	−51945.2	86,354.4	1.319	0.73	1176.1	2.418	1.842	2.308	1.915
12	5.347	−53014.6	88,771.4	1.425	0.753	1188.6	1.611	1.877	1.704	1.824
13	5.426	−49932.6	83,218.3	2.558	0.664	1143.3	1.902	1.940	1.961	1.947
14	5.574	−47793.5	74,926.6	−1.955	0.53	1119.3	1.927	1.985	1.942	2.093
16	5.465	−46288.2	74,260.7	9.347	0.874	1098.8	1.623	1.708	1.591	1.632
17	5.391	−55513.4	103,363.9	−4.042	0.744	1153.3	1.817	1.399	1.743	1.554
18	5.394	−52431.4	97,285.7	−4.294	0.685	1107.7	1.167	1.003	1.148	1.088
19	5.483	−50292.4	88,485.6	−7.332	0.519	1079.5	1.201	1.291	1.260	1.246
21	5.351	−56489.4	105,348.6	−2.732	0.826	1157.2	1.346	1.448	1.253	1.517
22	5.475	−53407.4	97,851.6	−2.68	0.753	1111.5	1.473	1.563	1.516	1.606
23	5.547	−51268.4	88,785.8	−6.024	0.603	1083.3	1.595	1.727	1.723	1.780
24	5.675	−52305	94,806.5	−6.472	0.736	1103	1.001	1.386	0.984	0.970
25	5.363	−55420	102,004.1	−2.701	0.801	1142.4	1.683	1.460	1.661	1.506
26	5.28	−56582.9	108,704.5	−4.016	0.768	1169.1	0.719	0.989	0.737	0.847
28	5.461	−51361.9	92,126.6	−7.315	0.544	1093.1	1.194	1.246	1.194	1.199
29	5.574	−52398.5	97,419.4	−7.874	0.675	1111.5	0.96	0.921	1.027	1.146
30	5.425	−57558.9	107,184.7	−2.663	0.849	1173	1.717	1.786	1.669	1.630
31	5.438	−54476.9	101,111	−2.977	0.787	1127.1	1.605	1.419	1.593	1.525
32	5.538	−52337.9	92,059.3	−6.003	0.626	1096.9	2.215	1.751	2.098	1.777
34	5.371	−56489.5	105,092	−2.767	0.822	1157.2	1.252	1.528	1.429	1.600

**Table 5:** Comparison between observed and predicted activities obtained using Y-randomization method.

*N*	3	11	19	26	29	31	33	2	7	9	18	20	22	24
Log IC_50_	2.367	2.418	1.201	0.719	0.96	1.605	1.603	1.496	1.405	1.193	1.167	0.897	1.473	1.001
Pred log IC_50_	2.124	1.813	1.294	0.834	0.882	1.439	1.458	1.637	1.604	1.706	1.009	0.843	1.612	1.442
*N*	30	27	34	8	14	15	4	1	12	5	13	23	25	17
Log IC_50_	1.717	0.953	1.252	2.368	1.927	2.13	1.701	1.958	1.611	1.5	1.902	1.595	1.683	1.817
Pred log IC_50_	1.706	1.125	1.489	2.084	1.982	2.259	1.686	1.614	1.828	1.646	1.932	1.800	1.470	1.300

**Table 6:** Comparison between experimental and predicted log IC_50_ values of the external test set for the MLR model based on descriptors of (3).

*N*	Log IC_50_	Pred log IC_50_	Residuals
5	1.500	1.565	−0.0400
9	1.193	1.589	0.4674
15	2.130	2.888	0.2912
20	0.897	0.517	−0.1386
27	0.953	0.842	0.0982
33	1.603	1.089	−0.6781

**Table 7:** Calculation of Golbraikh and Tropsha criteria.

Parameter	Formula	Threshold	Modelscore
*R*_ext_^2^	$R_{\text{ext}}^{2}=1-\left(\left(\sum {\left(Y_{\text{pred }\left(\text{test}\right)}-Y_{\left(\text{test}\right)}\right)}^{2}\right)/\left(\sum {\left(Y_{\text{test}}-\bar{Y_{\text{tr}}}\right)}^{2}\right)\right)$	*R*_ext_^2^ > 0.6>0.6	0.75
*r* ^2^	Coefficient of determination for the plot of predicted versus observed for the test set	*r* ^2^ > 0.6	0.77
*r*_0_^2^	*r* ^2^ at zero intercept		0.71
*r*′_0_^2^	*r* ^2^ for the plot of observed versus predicted activity for the test set at zero intercept		0.59
|*r*_0_^2^ − *r*′_0_^2^|		|*r*_0_^2^ − *r*′_0_^2^| < 0.3	0.12
*k*	Slope of the plot of predicted versus observed activity for the test set at zero intercept	0.85 < *k* < 1.15	1.07
(*r*^2^ − *r*_0_^2^)/*r*^2^		(*r*^2^ − *r*_0_^2^)/(*r*^2^) < 0.1	0.07
*k*′	Slope of the plot of observed versus predicted activity at zero intercept	0.85 < *k*′ < 1.15	0.92
(*r*^2^ − *r*′_0_^2^)/*r*^2^		(*r*^2^ − *r*′_0_^2^)/(*r*^2^) < 0.1	0.06
